# Editorial: Performance enhancement in rugby

**DOI:** 10.3389/fspor.2023.1212390

**Published:** 2023-05-09

**Authors:** F. Carson, T. Scott, M. Campo, M. J. Hamlin

**Affiliations:** ^1^ Department of Sport and Exercise Science, LUNEX International University of Health, Exercise and Sports, Differdange, Luxembourg; ^2^ Carnegie School of Sport, Leeds Beckett University, Leeds, United Kingdom; ^3^ Laboratoire de Psychologie, Dynamiques Relationnelles Et Processus Identitaires, Université de Bourgogne, Dijon, France; ^4^ Department of Tourism, Sport and Society, Lincoln University, Christchurch, New Zealand

**Keywords:** rugby union, rugby league, rugby sevens, concussion, physical characteristics, training & development, strength and conditioning, athlete wellbeing

**Editorial on the Research Topic** Performance enhancement in rugby

There has been a growth in performance related research in rugby union over the last decade, with much attention paid to the physical and psychological factors of performance (1, 2). Similarly, there has been an increase in scientific publications focusing on injury and injury prevention (3), and specifically on concussion (4). Our aim was to build on the existing scientific literature and further explore training, testing, and performance at the amateur and elite levels of rugby. This Research Topic of Frontiers in Sports and Active Living, “Performance Enhancement in Rugby” contains 6 original manuscripts that meet our aim.

Global participation rates in rugby (both league and union) appear to have reduced considerably due to the Covid pandemic and lack of opportunity to play. Despite this, women's and girl's playing rugby is soaring with World Rugby reporting a 28% increase in playing numbers since 2017, meaning 2.7 million female players are currently active (5). The increase in participation numbers has also led to an increase in scientific research in women's rugby. Within this Research Topic, two studies focus specifically on women's rugby. Ryan et al. have investigated the disclosure of concussion by female players, as female players appear to be more susceptible to concussion, take longer to recover and have more severe symptoms than male players. Their results suggest that a shared sense of team affiliation can help players engage with concussion-related education programs, which encourage greater disclosure of symptoms. The authors recommend practitioners to “explore how players can be incorporated into coaching and medical teams to encourage engagement with sport-related concussion education interventions and to improve communication and trust”. The second study investigated the development of physical characteristics of French women's rugby players over a 10-year period Imbert et al. The authors focused on 631 internationals from the French rugby fifteen a-side and rugby sevens, finding that the fifteen a-side players have improved strength and speed capabilities over this period. Meanwhile, players in the sevens format have witnessed increased improvements in their aerobic capacity and strength qualities, in addition to shifting anthropometrical characteristics (e.g., now taller and less fat mass). It is suggested that understanding this information can help adapt strength and conditioning programs to better prepare players for the demands of the game.

Three studies have concentrated on the physical demands of the game. Olsen et al. focused on the running demands of players at all levels of the fifteen a-side game in New Zealand. This is the first study that has compared these demands between high performance level (i.e., national and international level) and those at amateur / recreational level (i.e., club). Their findings somewhat contradict conventional thought that higher performance levels will have higher running demands, with the second lowest performance level recording the highest values in total distance and high intensity running. Further, the results indicate that there is considerable positional variation in running demands within and between competitions. Glaise et al. concentrated on the influence of repeated-sprint ability of semi-professional players, hypothesizing that repeated-sprint ability would correlate with key performance indicators during competition and be differentiated by position. Their findings indicate that higher repeated-sprint ability and the ability to reproduce these efforts has a significant impact on post-contact metres made, tackle breaks and tackles. The third study investigated the relationship between force-velocity profiles and match performance Heather et al. The authors looked at force-velocity profiles of 22 semi-professional male players and the connection between these and subsequent performance in games. The results suggest horizontal resistance training can improve tackling and tackle breaks, but that maximal power was not related to any key rugby performance indicator. This implies that training methods need to be more specified towards the activities within the game and that strength and conditioning coaches need to consider positional demands when designing resistance training programs.

Globally there has been an increased focus on professional athlete's mental health and wellbeing, with considerable resources being invested in this area. The final article in this Research Topic is related to the monitoring of player health and performance readiness by measuring neurological function and neuromuscular fatigue Daly et al. The authors discuss how many professional rugby teams have sought both subjective and objective measures in a bid to manage load and better prepare players for competition and reduce the incidence of injury. However, the authors argue that the use of non-invasive measurements of biopotentials (e.g., direct current potential; heart rate variability) may be better at determining a player's overall functional state. The findings suggest that combining these biopotentials with more traditional measures of player health and readiness may provide a more accurate assessment of the players health. The added benefit of non-invasively assessing neurological function and neuromuscular fatigue is that potentially useful changes can be detected, allowing for individual workloads to be managed.

At all levels of rugby, practitioners, coaches, and sport scientists are looking for strategies to improve performance. This Research Topic has added to the evidence-based practice on training, testing and performance methods, specific to the various codes of the game. The information can be used to develop best practice for monitoring physical and psychological performance and players readiness to participate. The included manuscripts contain information that may better prepare players for the physical demands of rugby and reduce the incidence of physical and brain injuries, while also considering player wellbeing.

## References

[1] Heyward O Nicholson B Emmonds S Roe G Jones B. Physical preparation in female rugby codes: An investigation of current practices. Front. Sports Act. Living. (2020) 2:584194. 10.3389/fspor.2020.584194 33345152 PMC7739696

[2] Kruyt N Grobbelaar H. Psychological demands of international rugby sevens and well-being needs of elite South African players. Front Psychol. (2019) 10:676. 10.3389/fpsyg.2019.00676 30984080 PMC6450168

[3] Paul L Naughton M Jones B Davidow D Patel A Lambert M Quantifying collision frequency and intensity in rugby union and rugby sevens: a systematic review. Sports Med Open. (2022) 8(1):12. 10.1186/s40798-021-00398-4 35050440 PMC8776953

[4] Brown JC Starling LT Stokes K Viviers P Jordaan E Surmon S High concussion rate in student community rugby union players during the 2018 season: implications for future research directions. Front Hum Neurosci. (2019) 13:423. 10.3389/fnhum.2019.00423 31866844 PMC6904273

[5] World Rugby. Women’s rugby (n.d.). Available at: https://www.world.rugby/organisation/about-us/womens?lang=en (Accessed April 20, 2023).

